# Teaching nutrition and sustainable food systems: justification and an applied approach

**DOI:** 10.3389/fnut.2023.1167180

**Published:** 2023-09-27

**Authors:** Christina Gayer Campbell, Gretchen Feldpausch

**Affiliations:** Department of Food Science and Human Nutrition, Iowa State University, Ames, IA, United States

**Keywords:** health, sustainable resilient healthy food and water systems, systems thinking, environment – agriculture, pedagogy, dietetics

## Abstract

Systems thinking is an essential skill for solving real-world problems, supporting lasting, impactful change, and creating desired futures. Transdisciplinary teaching and learning should be integrated into higher education to ensure students have the knowledge and skills to prosper in an ever-changing world. Education that addresses the interconnectedness of food systems is fundamental in cultivating future generations equipped to mitigate complex problems, such as hunger, nutrition-related chronic disease, and the climate crisis. Connecting the food, agriculture, and nutrition sectors is vitally important for improving human and planetary health and well-being. While we continue to acknowledge that it is critically important to teach systems thinking in the context of sustainable food systems limited resources are available to facilitate this type of learning. Historically, a “triple-bottom-line” approach focusing on economic, environmental, and social perspectives has been used to define sustainability. In contrast, including nutrition and health may provide a more robust view and even greater consideration for the system in its entirety. The sustainable, resilient, healthy food and water system framework, addressing all four pillars, can be used in higher education to help evaluate the sustainability of food and compare methods of production, place, and dietary patterns. This paper justifies the need for addressing sustainability issues in the context of nutrition and provides an educational approach to support student understanding and application of a systems thinking approach.

## 1. Introduction

Systems thinking is an essential skill for solving real-world problems, supporting lasting, impactful change, and creating desired futures (1). Various tools and strategies are available to help one cultivate a systems thinking perspective. For example, a system, defined as interacting, interrelated and interdependent components that form a complex and unified whole; requires one to shift from traditional linear ways of thinking. Fundamentally, this allows individuals to expand their current understanding of an issue and look at the bigger picture. Using this approach brings awareness to a greater number of contributing factors to a problem and, subsequently, various entry points for improvement and problem-solving. Systems thinking allows individuals to see how a change or shift in one part of the system can influence another interconnected part and helps evaluate the benefits, costs, and tradeoffs of different decisions made (2).

In regard to teaching and learning, utilizing systems thinking allows students to see things visually, organize their thoughts, change perspectives, and improve cross-disciplinary communication (1). It is well-suited to improve the use of, and adherence to, the precautionary principle, which takes preventative action and explores a wider range of alternatives to potentially harmful activities (3). It encourages the use of solutions with a diminished propensity to negatively influence the future. For example, identifying potential external consequences early in the problem-solving process to encourage more sustainable decision-making for the future health and well-being of people and the planet.

The food system is complex yet interconnected to various other important systems, for example, healthcare and natural biological systems (4). Furthermore, it is an essential component of contemporary education, given the current state of the world. Society is tasked with solving many exceedingly complex and precarious problems facing current and future populations; for example, the climate crisis, declines in human health and increase prevalence of chronic disease, hunger and malnutrition, and even the current reproductive crisis (5, 6). As we will address in upcoming sections, the food system is connected to the development of these problems; and thus should be carefully considered as we generate and implement solutions.

Research suggests that food systems are responsible for a third of global greenhouse gas emissions (7). Of the entire food supply chain, agriculture and land use were identified as the largest contributors at 71% of total emissions from the food system (7). Monoculture farming, growing one type of crop at one time in a specific area, is common in industrial agricultural systems (8). This practice is connected to diminished soil quality and increased erosion, both of which threaten future food production (9, 10). Furthermore, the increased application of synthetic fertilizers and agricultural chemicals creates environmental consequences for soil and water health and subsequent exposure to humans and animals (11). Along the food supply, approximately 40% of food being lost or wasted, contributing to a loss of resources required for production, as well as methane emissions associated with their breakdown in landfills (12).

The food system can also be linked to nutrient availability and human health. Nutrient density is defined as the ratio of nutrients to calories per bite of food. Although the nutrient composition of a food can vary based on geographic location, soil quality, and environmental conditions, generally speaking, there has been a decline in nutrient density of foods over time. One study compared nutrient composition in 43 garden crops between 1950 and 1999 using the USDAs nutrient profile data. They found a decline from 5 to 38 percent for various nutrients including protein, calcium, phosphorus, iron, riboflavin, and ascorbic acid (13). Furthermore, diets consumed today can be described as calorie-dense and nutrient-poor, particularly related to increased intake of ultra-processed foods which represents 73% of all food available to current US consumers (14). Poor diet quality is linked to increasing rates of non-communicable, chronic diseases such as obesity, diabetes, and heart disease (15). The current food system is directly connected to issues with consumer access and affordability of food, indicating that changes to the system will impact rates of hunger and malnutrition. Finally, human reproductive epidemiologists have identified an aggressive decline in male and female fertility, attributing the trend to lifestyle factors, including poor diet quality, and exposure to environmental contaminants, many of which are directly linked to the food system (16, 17).

Transdisciplinary teaching and learning should be integrated into higher education to ensure students have the knowledge and skills to prosper in an ever-changing world (18). Education that addresses the interconnectedness of food systems is fundamental in cultivating future generations equipped to solve the complex problems we have previously discussed. However, our educational systems and strategies often support siloed learning. Although this strategy allows students to understand the basics of their field of study and scaffold to more complex material, in the pursuit of disciplinary learning, we often lose sight of how individual topics, and our overarching disciplines relate to larger systems. Moreover, identifying the complexities of systems may help increase students’ understanding of how decisions made in their field influence society.

Systems thinking can support enhanced understanding and positive change at the nexus of where systems meet and function. In contrast, limited use of this approach may create a lack of appreciation for the complexities associated with a given problem. For example, it is important for individuals studying nutrition to be educated on agriculture and food production, separate fields of study. Connecting the food and agriculture sector to nutrition interventions has been discussed as a critical characteristic for improving hunger and food insecurity (19). Food systems models visually identify the complex interconnectedness of food, agriculture, and human nutrition (4). Given these relationships, issues such as the lack of sustainability guidance in nutrition recommendations can be problematic.

The Dietary Guidelines for Americans (DGAs) are recommendations published every 5 years by an expert panel of nutrition and dietetic professionals and regulated by the US Department of Health and Human Services and the US Department of Agriculture. They provide advice on what to eat and drink to meet our nutritional needs, promote health, and prevent diseases (20). Guidelines are written for professionals, policymakers, educators, and healthcare providers. Additionally, they function as guidelines for food programs in the United States; for example, nutrition recommendations for youth are the foundation for school breakfast and lunch programs (21).

Although the primary function of these recommendations is to promote healthy dietary patterns, the guidelines should consider the food system as a whole. Unfortunately, these guidelines do not address sustainability (22). Given this gap, there is a risk of system-altering recommendations that do not support a healthy food system for the future. Food production, transportation, processing, and consumption contribute to environmental sustainability and other societal issues previously discussed.

To illustrate, the DGAs currently recommend the consumption of 8–10 oz. of fish per person per day (20). However, the average seafood intake in the US is 5.6 ± 3.0 oz.; 80–90% of Americans do not meet this daily recommendation (23). The Food and Agriculture Organization of the United Nations state that one-third of the world’s assessed fisheries are being pushed beyond their biological limit and populations are rapidly declining due to overfishing (24). If all US consumers adhered to current nutrition recommendations, we would likely see even greater negatives consequences on fish populations. Aquaculture helps fill the gap in production and nutritional demand. However, this solution may also create unintended consequences that are of concern in the context of sustainability; for example, concentrated fish waste or increased disease risk associated with crowded conditions. As a second example, DGAs encourage consumers to increase intake of fruits and vegetables to support health and prevent chronic disease (20). However, to achieve this dietary goal at the population level, it would require change across the food supply chain, including shifts in food production and land use, adjustments to government policy, consumer buy-in, and overall greater consideration for the complex nature of food environments, including the 5 dimensions of food access (availability, accessibility, affordability, accommodation, acceptability) (25, 26).

Achieving the recommendations outlined in the DGAs would require changes to the current food system or, alternatively, create negative downstream consequences. Instead, policies and guidelines should consider the system as a whole and be provided only after weighing benefits and drawbacks from multiple perspectives, carefully considering future sustainability. Guidelines may also consider providing relevant information regarding the environmental impact of foods to support the consumer and stakeholder decision-making process. In addition, given previous examples, one might consider providing advice on what to look for when shopping for fish; or the benefits of buying local produce. Although sustainability in the DGAs is one example, it illustrates the potential consequences of non-systems-based approaches. As the example suggests, the nexus of nutrition, agriculture, and the environment is often ambiguous. As a result, decisions are made in silos without consideration for the system in its entirety, and consumers are left to navigate decision-making with conflicting information. Instead, food systems frameworks that support systems thinking can be used in educational settings to ensure future professionals are equipped with the skills to create positive systems change that acknowledges these intricacies.

Historically, a “triple-bottom-line” approach focusing on economic, environmental, and social perspectives has been used to encourage systems thinking in various disciplines (27). In contrast, a four-pillar method may provide a more robust view and even greater consideration for the system as a whole. The Sustainable, Resilient, Healthy Food and Water System (SRHFWS) framework can support the use of systems thinking in the context of food (28). This method considers nutrition and health, in addition to environmental stewardship, social, cultural, and ethical capital, and economic vitality. The framework can be used in various capacities and in conjunction with different food system models. More specifically, this framework can be used and applied in higher education to help critically evaluate the sustainability of individual food products, compare foods with varying methods of production and processing, those grown in different geographic locations, as well as to compare whole dietary patterns. The remainder of this paper will focus on detailing the four-pillar framework providing tangible examples for use in higher education.

## 2. Application

### 2.1. A four pillar method of analysis

**Figure 1:**
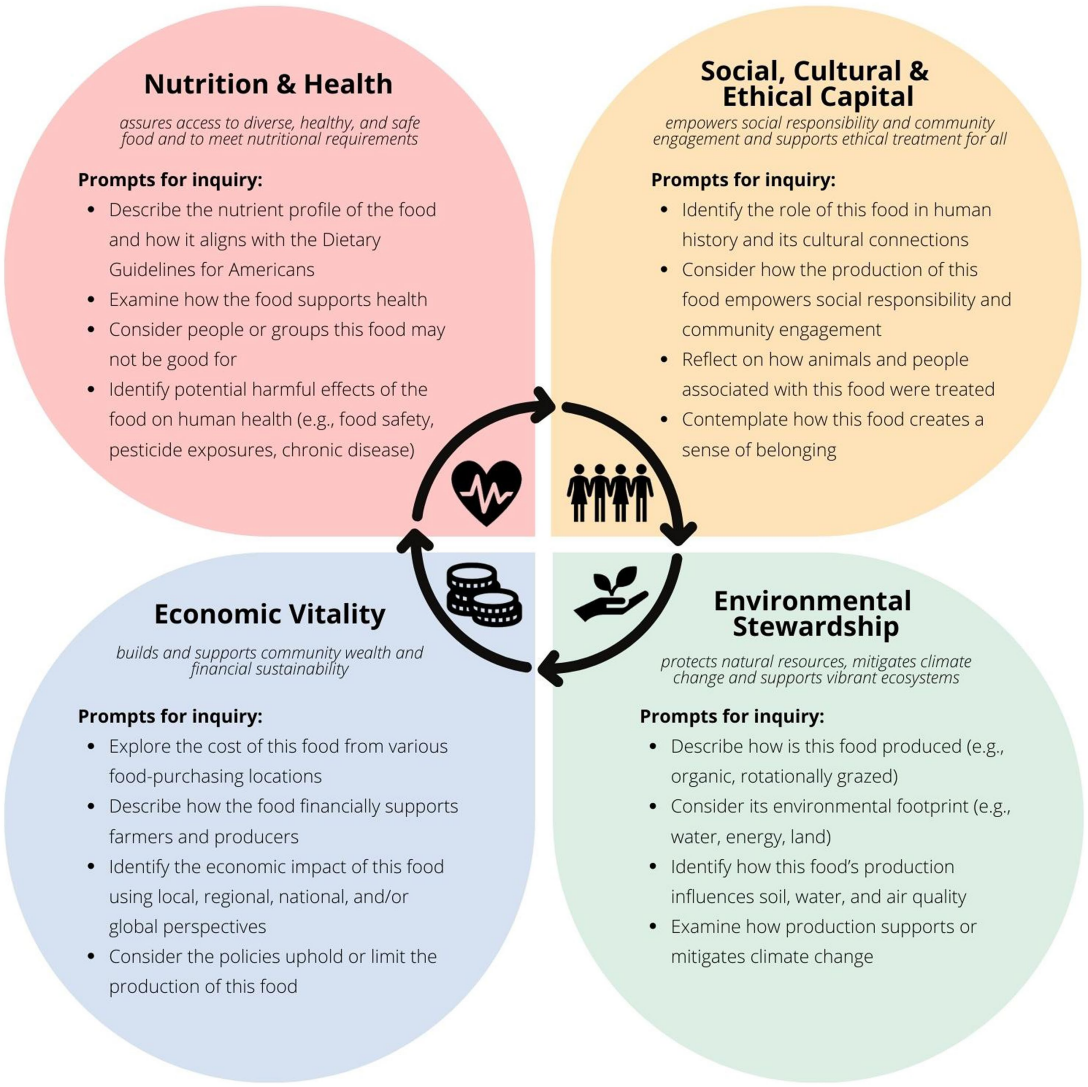
Four pillar method of analysis. Prompts to consider when evaluating characteristics of a food product using the sustainable, resilient, healthy food and water system framework (26).

Using the SRHFWS framework, Figure 1 provides pillar-specific prompts for students to consider when exploring the overall sustainability of a food or product. The initial step of choosing a food to explore provides an opportunity to discuss the diversity of food options and how this impacts sustainability. The food chosen for analysis should be specific in terms of its type, as well as how and where it was produced and processed. Then, accumulating evidence and perspective from each individual pillar allows students the opportunity to identify benefits and drawbacks before considering the interconnectedness of information gleaned from each pillar. The following sections outline the pillars and offer insight on how students might explore each area. In addition to the resources cited in the forthcoming descriptions of the four pillars; the *Food Systems Dashboard*, *Our World in Data*, and the *Center for Science in the Public Interest* may be beneficial references to consider for multiple pillars (29–31).

#### 2.1.1. Nutrition and health

The first component of the SRHFWS framework provides a nutrition and health perspective. A sustainable food system in this context “assures access to diverse, healthy, and safe food that meets nutritional requirements” (28). This pillar is a unique feature of this framework as most models of sustainability consider health a social construct. Addressing nutrition and health as a separate category allows for a deeper appreciation of the nutritional value and health implications of the food or system being considered. For example, one could contemplate the nutrition and health characteristics of a single food; for example, a potato. Students might consider investigating the nutritional profiles of foods (32), for example, the level of potassium, a shortfall nutrient in the American diet, provided by the potato. They may also contemplate how the foods are recommended to the public (20). The potato, to illustrate, is often excluded as a health food because it is primarily consumed in the United States in processed forms including French fries and potato chips; yet, as a whole food it is quite nutrient-dense. Student should also review literature on how the food supports health (33), and potential negative implications the food has on human health (34, 35).

#### 2.1.2. Social, cultural, and ethical capital

The second component addresses social, cultural, and ethical perspectives. This pillar represents the importance of a food system that “empowers social responsibility and community engagement and supports ethical treatment for all” (28). Continuing with the potato example, students might now explore the history of the potato, identify where the potato originated, and how it was introduced to different cultures around the world. Perhaps, a historical event is of interest, such as The Great Hunger. Depending on the academic level of students (e.g., undergraduate, graduate, professional), the depth of this investigation might vary. Students could simply define The Great Hunger or explore the role of government and policy in how the conflict arose. Community engagement might be a relevant topic particularly for places who grow the food. How are individuals in the community engaged with the production of this food? Where is the product primarily grown or produced? Where is this place in relationship to where your students live? Are there any ethical issues associated with growing, producing, harvesting this food product for either humans or animals? In what form do different cultures consume the food? Finally, students should consider how this product promotes a sense of belonging which speaks to the cultural importance of the food. Students might explore different cultural connections associated with the food product; for example, its use in a family recipe, an interview with family or community members, or an internet search using key terms for the food and how different regions around the globe utilize this food.

#### 2.1.3. Environmental stewardship

The next component, environmental stewardship, is the third pillar of the SRHFWS framework. A sustainable food system from this lens “protects natural resources, mitigates climate change and support vibrant ecosystems” (28). Items for consideration in this pillar might address overall the environmental footprint of this food across the supply chain (36). Specifically, what land, water, and energy resources were required at various stages of its production? For example, potatoes have a lower carbon footprint and require less land and water in production than many other fruits, vegetables, and cereals. Students might reflect on what fertilizers and pesticides are used and their implications. What practices or regulations were followed in production (e.g., industrial, regenerative)? Is this food product on the Environmental Working Group Dirty Dozen™ list? (37). Based on the footprint, are there any known impacts on soil, water, or air quality as a result of how this food was grown? Does the production strategy promote soil health or mitigate negative environmental consequences such runoff? Growing potatoes, for example, creates a great deal of physical disruption to the soil, yet specific strategies such as reduced tillage may mitigate the negative consequences. What is the role of this food as it relates to climate conditions including the amount of greenhouse gas emissions released across the supply chain? (38, 39). Finally, students might consider how the food product relates to the issue of food waste (36).

#### 2.1.4. Economic vitality

The final SRHFWS pillar addresses economic vitality; whereby products and systems “build and support community wealth and financial sustainability” (28). A simple approach to addressing this pillar includes a cost analysis at different food purchasing locations. Students can “shop online” for foods from a variety of stores such regionally owned supermarkets. Alternatively, students can visit cooperatives, dollar stores, convenience stores, or supercenters to compare costs. Are the foods available and appropriately priced at all of these places? The potato, for example, is often considered a budget-friendly food option. One might also consider if SNAP or WIC benefits cover the cost for the consumer. Does the purchasing location financially support a local farmer? If the food does not support health, what are the long-term economic implications associated with increased disease risk for individuals and the larger society? For example, what is the “cost” on consumer health from consuming potatoes whole? As potato chips? Finally, the economic pillar provides an opportunity to address whether there are policies in place that impact how this food is grown (e.g., farm subsidies). This may include local policies, or federal policies such as the Farm Bill. Perhaps the food is a commodity and therefore has economic implications on the producer.

#### 2.1.5. Creating connections

The process of responding to prompts of inquiry for each pillar is inherently siloed. Thus, before moving to an advanced application, students should describe how the information that surfaced during their analysis is interrelated. To illustrate, one might consider the story of chemical use given the potato example we have described thus far. Pesticide and herbicide application is often used to limit pest and weed pressure that could negatively impact yield; which is essential to ensure farmers are supported economically. In addition, some chemicals are used to help the desiccation of vines on potatoes, a common pre-harvest practice that helps with controlling tuber size and protecting against some viruses. However, we see various downstream effects. Chemical inputs such as these persist in the soil, air, and water. Air and water pollution can influence the health of farmers applying such chemicals, as well as those in close proximity to farms. Soil pollution may also influence the sustainability of producing food on that land in the future as heavy treatment can cause a decline in beneficial microorganisms. The chemicals may end up on the food products themselves and, if not processed and cleaned effectively, can increase the amount of residue ingested by consumers. Such exposures subsequently can increase risk of diseases and cancers depending on the chemical. One might finally consider whether these potential environmental and health consequences are ethical for producers, consumers, and animals. This is but a single example of how one topic, chemical application, is interconnected among the pillars. Students should consider viewing the information gleaned from the lens of each pillar and consider how those topics relate to other pillars. During this process, students may identify gaps in their analysis whereby answering new questions my provide a more robust understanding of the food/product.

### 2.2. Advanced application using production, place, and pattern

**Figure 2:**
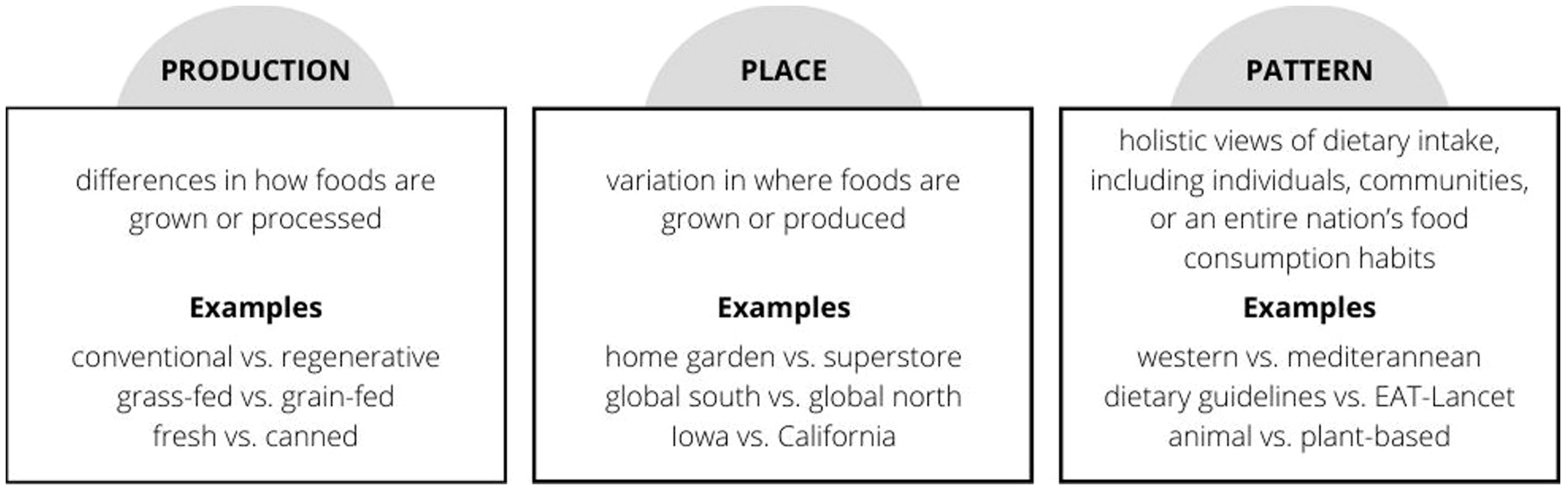
Advanced application. The four pillar method of analysis can be further applied, in a more advanced setting, by comparing foods with varying characteristics such as those with different production or processing methods, or those grown in different geographic locations. The third, and most advanced application might include a four pillar analysis and comparison of whole dietary patterns.

As students move from novice to competent in their ability to evaluate a single food product using a four-pillar method (Figure 1), more complex scenarios can be introduced. Figure 2 visually depicts how we might consider comparing the sustainability of two food-related topics. More specifically, one might compare foods that vary in method of production or processing, or foods grown or raised in different places. To further advance the evaluation, one may also consider comparing two food patterns, for example, the Western vs. Mediterranean diets. Instructors should consider using a guided activity, such as that provided in Supplementary material S1, to provide structure for students working through this process.

*Production* includes how food is grown, processed, or prepared. For example, students can evaluate a food grown using conventional or industrial agricultural practices versus being grown under organic or regenerative conditions, or the differences in a whole food versus processed option; for example a potato versus potato chips. Many of the prompts of inquiry provided in Figure 1 can be used for this comparative analysis. In this situation, students should be encouraged to reflect on the similarities and differences for each pillar and highlight which of the four pillars appears to be impacted the most given the variation in the two products.

*Place* allows students the opportunity to compare how a foods origin influences sustainability. For example, considering how a food grown in a garden, at a local farm, transported across a country, or imported changes characteristics in each pillar. Perhaps this influences the economic or environment pillars due to differences in transportation or variation in soil composition in different places creates differences in nutrient density. Expanding on the potato example we have used thus far, one might consider potatoes grown in Idaho versus those imported from China; or whether it is more sustainable to grow them in the Northwest versus Southeast United States? Coming from the lens of *place*, we are also able to consider foods traditionally grown in the area of interest. What is indigenous to this land? What were the foods eaten in this place prior to industrialization of the food system? This application scenario encourages students to delve into foods grown in different regions within their country of residence or compare different countries. The *Food Systems Dashboard* may be particularly helpful as a resource in this form of evaluation (29).

*Patterns* provide more holistic perspective of diet rather than focusing on a single food. Dietary patterns are defined as “the quantities, proportions, variety, or combination of different foods, drinks, and nutrients in diets, and the frequency with which they are habitually consumed” (20). Consider the current eating patterns of a specific population as reflected in national recommendations (20, 40, 41). How do current recommendations for sustainable ways of eating align with food availability and access in specific places? For example, do the EAT-Lancet, plant-forward recommendations work for people living in specific places such as the Global South or places in the far north? (42). Evaluating patterns is the most advanced form of application when considering the strategies we have presented.

## 3. Discussion

Utilizing a four-pillar approach provides students with an opportunity to understand complexities in the food system, a necessary skill for solving real-world problems. Students should be encouraged to consider foods from the perspective of each individual pillar as well as acknowledge their interconnectedness. Completing a more in-depth analysis can be done by comparing different products based on production and processing practices, place, and overall dietary patterns. This provides a more robust assessment of the food system and acknowledges the benefits and drawbacks of various food choices in the context of sustainable, healthy diets. Students should be prompted to reflect on how this analysis supports their ability to see the big picture and learn from new perspectives, both key characteristics of systems thinking.

## Author contributions

CC and GF contributed equally to the conception, design, writing, and editing of the manuscript. All authors have read and agreed upon the work submitted.

## References

[1] Waters Center for Systems Thinking. Why systems thinking? (2023). Available at: https://waterscenterst.org/why-systems-thinking?tab=benefits (Accessed February 2, 2023).

[2] The Systems Thinker. Systems thinking: what, why, when, where, and how? (2018). Available at: https://thesystemsthinker.com/systems-thinking-what-why-when-where-and-how/ (Accessed February 2, 2023).

[3] Kriebel D Tickner J Epstein P Lemons J Levins R Loechler EL. The precautionary principle in environmental science. Environ Health Perspect. (2001) 109:871–6. doi: 10.1289/ehp.01109871, PMID: 11673114 PMC1240435

[4] Nourish. Food system tools. (2023). Available at: https://www.nourishlife.org/teach/food-system-tools/ (Accessed February 2, 2023).

[5] The Lancet. The global syndemic of obesity, undernutrition and climate change: the Lancet comission report. (2019) Available at: https://www.foodpolitics.com/wp-content/uploads/ObesityCommission_Policy-Brief_Lancet_19.pdf (Accessed February 2, 2023). 10.1016/S0140-6736(18)32822-8 30700377

[6] Sutton P Wallinga D Perron J Gottlieb M Sayre L Woodruff T. Reproductive health and the industrialized food system: a point of intervention for health policy. Health Aff. (2011) 30:888–97. doi: 10.1377/hlthaff.2010.1255, PMID: 21555472 PMC6693635

[7] Crippa M Solazzo E Guizzardi D Monforti-Ferrario F Tubiello FN Leip A. Food systems are responsible for a third of global anthropogenic GHG emissions. Nat Food. (2021) 2:198–209. doi: 10.1038/s43016-021-00225-9 37117443

[8] Natural Resources Defense Council. Industrial agriculture. (2020). Available at: https://www.nrdc.org/stories/industrial-agriculture-101#monoculture (Accessed February 2, 2023).

[9] Sustainable Agriculture Research and Education. Building soils for better crops: ecological management for healthy soils. (2021). Available at: https://www.sare.org/resources/building-soils-for-better-crops/ (Accessed February 2, 2023).

[10] Our World in Data. Do we only have 60 harvests left? (2021). Available at: https://ourworldindata.org/soil-lifespans (Accessed February 2, 2023).

[11] UN Environment Programme. Report: environmental and health impacts of pesticides and fertilizers and ways of minimizing them. (2021). Available at: https://www.unep.org/resources/report/environmental-and-health-impacts-pesticides-and-fertilizers-and-ways-minimizing (Accessed February 2, 2023).

[12] Natural Resource Defense Council. Report – wasted: how America is losing up to 40% of its food from farm to fork to landfill. (2017). Available at: https://www.nrdc.org/sites/default/files/wasted-2017-report.pdf (Accessed February 2, 2023).

[13] Davis DR Epp MD Riordan HD. Changes in USDA food composition data for 43 garden crops, 1950 to 1999. J Am Coll Nutr. (2004) 23:669–82. doi: 10.1080/07315724.2004.10719409, PMID: 15637215

[14] Ravandi B Mehler P Barabási AL Menichetti G. GroceryDB: prevlaence of processed food in grocery stores. medRxiv [Preprint]. (2022). doi: 10.1101/2022.04.23.22274217v2 PMC12579494 39806219

[15] Fanelli SM Jonnalagadda SS Pisegna JL Kelly OJ Krok-Schoen JL Taylor CA. Poorer diet quality observed among US adults with a greater number of clinical chronic disease risk factors. J Prim Care Community Health. (2020) 11:2150132720945898. doi: 10.1177/2150132720945898, PMID: 32996366 PMC7533933

[16] Sun H Gong TT Jiang YT Zhang S Zhao YH Wu QJ. Global, regional, and national prevalence and disability-adjusted life-years for infertility in 195 countries and territories, 1990-2017: results from a global burden of disease study, 2017. Aging. (2019) 11:10952–91. doi: 10.18632/aging.102497, PMID: 31790362 PMC6932903

[17] Skakkebæk NE Lindahl-Jacobsen R Levine H Andersson AM Jørgensen N Main KM. Environmental factors in declining human fertility. Nat Rev Endocrinol. (2022) 18:139–57. doi: 10.1038/s41574-021-00598-8 34912078

[18] Krettek A Thorpenberg S. Transdisciplinary higher education—a challenge for public health science. Educ Res Int. (2011) 2011:649539:1–6. doi: 10.1155/2011/649539

[19] Duncan E Ashton L Abdulai AR Sawadogo-Lewis T King SE Fraser EDG. Connecting the food and agriculture sector to nutrition interventions for improved health outcomes. Food Sec. (2022) 14:657–75. doi: 10.1007/s12571-022-01262-3, PMID: 35126795 PMC8804081

[20] USDA. Dietary guidelines for Americans 2020–2025. (2020). Available at: https://www.dietaryguidelines.gov/sites/default/files/2020-12/Dietary_Guidelines_for_Americans_2020-2025.pdf (Accessed February 2, 2023).

[21] Federal Registrar. Child nutrition programs: revisions to meal patterns consistent with the 2020 dietary guidelines for Americans. (2023). Available at: https://www.federalregister.gov/documents/2023/02/07/2023-02102/child-nutrition-programs-revisions-to-meal-patterns-consistent-with-the-2020-dietary-guidelines-for (Accessed February 8, 2023).

[22] US Department of Agriculture. 2015 dietary guidelines: giving you the tools you need to make healthy choices. (2017). Available at: https://www.usda.gov/media/blog/2015/10/06/2015-dietary-guidelines-giving-you-tools-you-need-make-healthy-choices (Accessed February 2, 2023).

[23] Jahns L Raatz SK Johnson LK Kranz S Silverstein JT Picklo MJ Sr. Intake of seafood in the US varies by age, income, and education level but not by race-ethnicity. Nutrients. (2014) 6:6060–75. doi: 10.3390/nu6126060, PMID: 25533013 PMC4277015

[24] Food and Agriculture Organization. The state of the world fisheries and aquaculture. (2022). Available at: https://www.fao.org/3/cc0461en/online/sofia/2022/status-of-fishery-resources.html (Accessed February 2, 2023).

[25] Nesheim MC Oria M Tsai YP. A framework for assessing effects of the food system. Washington DC: National Academies Press (2015). 26203480

[26] Caspi CE Sorensen G Subramanian SV Kawachi I. The local food environment and diet: a systematic review. Health Place. (2012) 18:1172–87. doi: 10.1016/j.healthplace.2012.05.006, PMID: 22717379 PMC3684395

[27] Purvis B Yong M Robinson D. Three pillars of sustainability: in search of conceptual origins. Sustain Sci. (2019) 14:681–95. doi: 10.1007/s11625-018-0627-5

[28] Spiker M Reinhardt S Bruening M. Academy of nutrition and dietetics: revised 2020 standards of professional performance for registered dietitian nutritionists (competent, proficient, and expert) in sustainable, resilient, and healthy food and water systems. J Acad Nutr Diet. (2020) 120:1568–1585.e28. doi: 10.1016/j.jand.2020.05.010, PMID: 32829776

[29] Food Systems Dashboard (2023). Available at: https://www.foodsystemsdashboard.org/ (Accessed February 2, 2023).

[30] Our World in Data (2023). Available at: https://ourworldindata.org/ (Accessed February 2, 2023).

[31] Center for Science in the Public Interest (2023). Available at: https://www.cspinet.org/ (Accessed February 2, 2023).

[32] US Department of Agriculture. FoodData Central. (2019). Available at: https://fdc.nal.usda.gov/ (Accessed February 2, 2023).

[33] National Library of Medicine. PubMedgov. (2023). Available at: https://pubmed.ncbi.nlm.nih.gov/ (Accessed February 2, 2023).

[34] Environmental Working Group. Know your choices. (2023). Available at: https://www.ewg.org/ (Accessed February 2, 2023).

[35] Agriculture Marketing Service of the US Department of Agriculture. Pesticide data program. (2021). Available at: https://www.ams.usda.gov/datasets/pdp (Accessed February 2, 2023).

[36] Environmental Protection Agency. From farm to kitchen: the environmental impacts of U.S. food waste (2021). Available at: https://www.epa.gov/system/files/documents/2021-11/from-farm-to-kitchen-the-environmental-impacts-of-u.s.-food-waste_508-tagged.pdf (Accessed February 2, 2023).

[37] Environmental Working Group. Shoppers guide to pesticides in produce. (2022). Available at: https://www.ewg.org/foodnews/dirty-dozen.php (Accessed February 2, 2023).

[38] Poore J Nemecek T. Reducing food’s environmental impacts through producers and consumers. Science. (2018) 360:987–92. doi: 10.1126/science.aaw9908 29853680

[39] Our World in Data. The carbon footprint of foods: are differences explained by the impacts of methane? (2020). Available at: https://ourworldindata.org/carbon-footprint-food-methane (Accessed February 2, 2023).

[40] Government of Canada. Canada’s food guide. (2023). Available at: https://food-guide.canada.ca/en/ (Accessed February 2, 2023).

[41] Australian Government. The Australian dietary guidelines. (2019). Available at: https://www.health.gov.au/resources/publications/the-australian-dietary-guidelines (Accessed February 2, 2023).

[42] Eat Lancet. EAT-Lancet commission summary report (2019). Available at: https://eatforum.org/eat-lancet-commission/eat-lancet-commission-summary-report/ (Accessed February 2, 2023).

