# Supplementary material for: Teaching nutrition and sustainable food systems: justification and an applied approach
Source: Front Nutr. 2023 Sep 27;10:1167180. doi: 10.3389/fnut.2023.1167180 (PMC10564979; doi:10.3389/fnut.2023.1167180)
Supplement: Supplementary file 1 [file Data_Sheet_1.pdf]

Sustainability Analysis: Advanced Application Worksheet Template

Purpose: Analyze the sustainability of foods, products, or dietary patterns using a 4-pillar approach

Aim: Facilitate systems thinking in the context of making sustainable food choices

Objectives:

1. Analyze two foods, products, or dietary patterns using a 4-pillar approach (nutrition & health; social, cultural & ethical capital; environmental stewardship; and economic vitality)
2. Explore the interconnectedness of information gleaned from each pillar
3. Identify and justify which option supports a more sustainable, resilient, healthy food system
4. Reflect on how your (1) perspective and (2) understanding changed in the context of the larger food system

Part A: Pre-Analysis Reflection

1. What is your chosen food/product or dietary pattern?

____

2. What type of comparative analysis are you planning on conducting?

- ☐ Production (e.g., organic vs. conventionally grown food)
- ☐ Place (e.g., food grown locally vs. imported from overseas)
- ☐ Pattern (e.g., Mediterranean vs. Western)

3. Given your chosen food/product or pattern (#1) and the type of comparative analysis you are conducting (#2), what two options do you plan to compare?

Option 1:

Option 2:

*You will now move to **Part B**, your comparative analysis.*
Use the prompts provided here to guide your thinking as you explore.
Be sure to record the literature and resources used in your analysis.

	**Prompts to Consider**
**Nutrition & Health**	1. Describe the nutrient profile of the food and how it aligns with the Dietary Guidelines for Americans<br>2. Examine how the food supports one's health<br>3. Consider people or groups this food may not be good for<br>4. Identify potential harmful effects of the food on human health (e.g., food safety, pesticide exposures, chronic disease)
**Social, Cultural & Ethical Capital**	1. Identify the role of this food in human history and its cultural connections<br>2. Consider how the production of this food empowers social responsibility and community engagement<br>3. Reflect on how animals and people associated with this food were treated<br>4. Contemplate how this food creates a sense of belonging
**Environmental Stewardship**	1. Describe how is this food produced (e.g., organic, rotationally grazed)<br>2. Consider its environmental footprint (e.g., water, energy, land)<br>3. Identify how this food's production influences soil, water, and air quality<br>4. Examine how production supports or mitigates climate change
**Economic Vitality**	1. Explore the cost of this food from various food purchasing locations<br>2. Describe how the food financially supports farmers and producers<br>3. Identify the economic impact of this food using local, regional, national and/or global perspectives<br>4. Consider the policies uphold or limit the production of this food

Part B: Comparative Analysis

Use the spaces below to record relevant information associated with each pillar for both options in your analysis. Prompts on the previous page can be used to guide your research. **Note:** Additional information and exploration may be needed based on your chosen food/product or pattern.

	**Option A: ___________________________________**	**Option B: ___________________________________**
**Nutrition & Health**		
**Social, Cultural & Ethical Capital**		

**Environmental Stewardship**		
**Economic Vitality**		

Part C: Post-Analysis Reflection

1. Creating Connections. *The four-pillar approach provides a structured way to consider the complex nature of a specific food/product or pattern. Once your analysis is complete, explore connections between the four pillars and consider how your findings intertwine. How does a characteristic from one pillar interconnect to those in another? Does one characteristic lead to changes (both positive or negative) in other pillars?*

2. Making Informed Decisions. *Using the information you've collected and assessing the relationships between the pillars, in your opinion, which option supports a more sustainable, resilient, healthy food system? Explain.*

3. Cultivating New Perspectives. *Considering all you have learned through this process, reflect on how your perspective of this food/product has changed.*

4. Utilizing a Systems Approach. *After conducting your analysis, consider potential upstream and downstream consequences of your food/product or dietary pattern. (Note: Use a relevant food system model, map, or framework to guide your reflection)*

